# Nutritional Interventions in Head and Neck Cancer Patients Undergoing Chemoradiotherapy: A Narrative Review

**DOI:** 10.3390/nu7010265

**Published:** 2015-01-05

**Authors:** Maurizio Bossola

**Affiliations:** Department of Surgery, Catholic University, University Hospital “A. Gemelli”, Largo A. Gemelli, 8-00168 Rome, Italy; E-Mail: maubosso@tin.it; Tel.: +39-6-30155485; Fax: +39-6-30155491

**Keywords:** head and neck cancer, chemoradiotherapy, malnutrition, nutrition, nutritional counseling, oral nutritional supplements, enteral nutrition, gastrostomy

## Abstract

The present review aimed to define the role of nutritional interventions in the prevention and treatment of malnutrition in HNC patients undergoing CRT as well as their impact on CRT-related toxicity and survival. Head and neck cancer patients are frequently malnourished at the time of diagnosis and prior to the beginning of treatment. In addition, chemo-radiotherapy (CRT) causes or exacerbates symptoms, such as alteration or loss of taste, mucositis, xerostomia, fatigue, nausea and vomiting, with consequent worsening of malnutrition. Nutritional counseling (NC) and oral nutritional supplements (ONS) should be used to increase dietary intake and to prevent therapy-associated weight loss and interruption of radiation therapy. If obstructing cancer and/or mucositis interfere with swallowing, enteral nutrition should be delivered by tube. However, it seems that there is not sufficient evidence to determine the optimal method of enteral feeding. Prophylactic feeding through nasogastric tube or percutaneous gastrostomy to prevent weight loss, reduce dehydration and hospitalizations, and avoid treatment breaks has become relatively common. Compared to reactive feeding (patients are supported with oral nutritional supplements and when it is impossible to maintain nutritional requirements enteral feeding via a NGT or PEG is started), prophylactic feeding does not offer advantages in terms of nutritional outcomes, interruptions of radiotherapy and survival. Overall, it seems that further adequate prospective, randomized studies are needed to define the better nutritional intervention in head and neck cancer patients undergoing chemoradiotherapy.

## 1. Introduction

Head and neck cancer (HNC) (cancer of the oral cavity, oropharynx, hypopharynx and larynx) is the seventh most common malignancy in the world [1]. The majority of patients with HNC present with locally advanced disease [1], for which treatment is complex and aggressive, with a therapeutic goal of achieving a cure while minimizing toxicity. The standard of care is multidisciplinary, utilizing bimodality or trimodality therapy where appropriate. Recent advances have led to alterations in radiotherapeutic technologies, the introduction of sequential (induction) systemic chemotherapy, and the inclusion of targeted agents in combination chemotherapy regimens [2].

Head and neck cancer patients are frequently malnourished at the time of diagnosis and prior to the beginning of treatment [3,4,5,6,7]. In addition, chemo-radiotherapy (CRT) causes or exacerbates symptoms, such as alteration or loss of taste, mucositis, xerostomia, fatigue, nausea and vomiting, with consequent worsening of malnutrition [8,9,10,11,12]. It is well known that radiotherapy is invariably associated with mucositis, xerostomia, dysphagia, hematological toxicities and other acute side effects, whose incidence increases when chemotherapy is also administered, and that oral mucositis incidence leads to higher unplanned breaks and delays in radiotherapy administration [13,14,15,16]. In addition, in many patients such toxicities may be very severe and even life threatening and may lead to treatment interruptions that are invariably associated with poorer outcome [13,14,15,16]. To this regard, it has been shown that during radiotherapy or CRT 55% of the patients may lose an additional 10% or more of body weight [11,12]. Deterioration of the nutritional status results in an increase in CRT-related toxicity and this may increase the prolonged treatment time, which has been associated with poor clinical outcome [17,18].

In the current clinical practice, nutritional counseling with or without oral nutritional supplements patients receiving CRT for head and neck cancer is considered adequate [19] but its real role still remains to be clearly defined with regard to CRT-related toxicity.

If obstructing cancer and/or mucositis interfere with swallowing, enteral nutrition should be delivered by tube. However, it seems that there is not sufficient evidence to determine the optimal method of enteral feeding [19].

Prophylactic feeding through nasogastric tube or percutaneous gastrostomy has become relatively common. It remains to be defined if prophylactic feeding, compared to reactive feeding (patients are supported with oral nutritional supplements and when it is impossible to maintain nutritional requirements enteral feeding via a NGT or PEG is started), offers advantages in terms of nutritional outcomes, interruptions of radiotherapy and survival.

This review aimed to define the role of nutritional counseling, oral nutritional supplements, enteral nutrition through nasogastric tube or gastrostomy, and prophylactic gastrostomy in the prevention and treatment of malnutrition in HNC patients undergoing CRT as well as their impact on CRT-related toxicity and survival.

## 2. Methods

The following databases were searched for relevant studies up to October 2014: Medline, PubMed, Web of Science, and the Cochrane Library. The search terms and mesh headings included “head and neck neoplasms” OR “head and neck cancer” AND “nutrition” OR “nutrition support” OR “dietary counselling” OR “nutritional counselling” or “ nutritional supplements” OR “nutrition therapy” OR “gastrostomy” OR enteral nutrition” OR “enteral feeding” OR “prophylactic gastrostomy” OR “reactive gastrostomy” OR “prophylactic nutrition” OR “prophylactic nutritional support”. Reference lists of relevant studies and previous systematic reviews were manually searched for additional articles.

Studies were eligible for inclusion if they were English language papers published in a peer-reviewed journal and met the following inclusion criteria: primary research studies in adult patients (over 18 years of age), included patients with head and neck cancer receiving radiotherapy or radiochemotherapy as the primary treatment, and investigated nutritional interventions in the form of oral supplements or dietary counseling or both or enteral nutrition through nasogastric tube or gastrostomy with primary outcomes, including dietary intake, weight, nutritional status, quality of life, functional status, treatment response, radiotherapy toxicity, or survival. None of the studies with such characteristics was excluded.

## 3. Nutritional Counseling and Oral Nutritional Supplements

International guidelines suggest that intensive nutritional counseling (NC) and oral nutritional supplements (ONS) should be used to increase dietary intake and to prevent therapy-associated weight loss and interruption of radiation therapy in patients undergoing radiotherapy or chemoradiotherapy of head and neck areas [20,21,22].

This evidence is based essentially on a randomized study performed in 60 oncology outpatients receiving radiotherapy to the gastrointestinal (12%) or head and neck areas (78%). This study documented statistically smaller deteriorations in weight, nutritional status and global quality of life when intensive, individualized NC and ONS were used instead of standard nutritional care [4,5,21]. Indeed, Ravasco *et al.* [23], two years earlier, randomized 75 HNC patients referred for radiotherapy/chemoradiotherapy to dietary counseling with regular foods (Group 1), usual diet plus supplements (Group 2) and food intake *ad libitum* (Group 3). At three months, Group 1 maintained intakes, whereas Groups 2 and 3 returned to or below baseline levels. In addition, at three months, the reduction of incidence/severity of symptoms (anorexia, nausea/vomiting, xerostomia, and dysgeusia) improved in 90% of the patients in Group 1 *vs.* 67% in Group 2 and 51% in Group 3 (*p <* 0.0001). QOL function scores improved (*p* < 0.003) proportionally with improved nutritional intake and status in Group 1/Group 2 (*p <* 0.05) and worsened in Group 3 (*p <* 0.05).

Thereafter, few studies have been conducted on this topic (Table 1). The recent prospective study of van den Berg *et al.* [24] has clearly confirmed that early and intensive individualized dietary counseling by a dietitian produces clinically relevant effects in terms of decreasing weight loss and malnutrition compared with standard nutritional counseling. All these data are in accordance with previous studies that have evaluated the effects of nutritional counseling and/or ONS in head and neck cancer undergoing radiotherapy only [25,26].

**Table 1:** Nutritional counseling (NC) and oral nutritional supplements (ONS) in head and neck cancer patients receiving radiotherapy (RT) or chemoradiotherapy (CRT).

Author	Number of Patients	Cancer Therapy	Nutritional Outcome	Interruption of RT
Arnold and Richter, 1989 [25]	Group 1: no nutritional supplements; Group 2: nutritional supplements	RT	No differences between the groups	No differences between the groups
Nayel *et al.*, 1992 [26]	Group 1: 12 pts; radiotherapy alone; Group 2: 11 pts; radiotherapy and ONS	RT	Group 1: in all increase in body weight and in triceps skin-fold thickness, Group 2: 58% had WL (*p =* 0.001)	Group 1: 41.6%; Group 2: 0%; (*p* < 0.001)
Goncalves Diaz *et al.*, 2005 [27]	Group 1: 32 pts; adapted oral diet; Group 2: 16 pts; enteral nutrition via a NG tube (6x/day); Group 3: 16 pts; oral diet associated to ONS between meals (3×/day).	RT	All of the groups presented an increase in the ingestion of calories and proteins (*p <* 0.001).	Not assessed
Ravasco *et al.*, 2005 [23]	Group 1: 25 pts; NC with regular foods; Group 2: 25 pts; usual diet with ONS; Group 3: 25 pts; intake *ad lib*.	CRT	Reduction of anorexia, nausea/vomiting, xerostomia, and dysgeusia: Group 1: 90% of pts; Group 2: 67% of pts; Group 3: 51% of pts	No differences among the groups
Isenring *et al.*, 2007 [21]	Group 1: 31 pts; standard practice; Group 2: 29 pts; individualized NC	CRT	Smaller deteriorations in weight, nutritional status and global quality of life in group 2	Not assessed
Paccagnella *et al.*, 2010 [28]	Group 1: 33 pts; early nutritional intervention before they were submitted to CRT; Group 2: 33 pts; CRT alone	CRT	Group 1: WL (%) 4.4 ± 4.2; Group 2: WL (%) 8.1 ± 4.8; (*p <* 0.01)	Group 1: 30.3%; Group 2: 63.6%; (*p <* 0.01)
Van den Berg, 2010 [24]	Group 1: 20 pts; individual dietary counseling; Group 2: 18 pts; standard dietary counseling	CRT	Group 1: WL (%) 2.3 ± 1.2; Group 2: WL (%) 4.8 ± 2.2	Not assessed
Valentini *et al.*, 2012 [29]	21 pts with NC and ONS	CRT	-	28% for ≥6 days, 28% for 3–5 days and 44% for 0–2 days

In the current clinical practice, NC with or without ONS in patients receiving CRT for head and neck cancer are considered useful but its real role still remains to be clearly defined with regard to CRT-related toxicity. It is possible, in fact, that the improvement of nutritional status obtained through NC and/or ONS may translate in reduced CRT-related toxicity. Unfortunately, data on this issue are few. Among the studies, which included patients receiving radiotherapy, CRT-related toxicity was not assessed in one study and assessed in two (with no differences in one study and with a beneficial effect of ONS on CRT-related toxicity in the other one). Among the studies which included patients receiving chemo-radiotherapy, three did not assess CRT-related toxicity, one found no differences in CRT-related toxicity between patients receiving or not receiving counseling/ONS, and two found such differences. Paccagnella *et al.* [28] showed that the frequency of grade 3–4 mucositis was 45.5% and 39.4% in patients with head and neck cancer undergoing concurrent chemoradiotherapy and receiving, respectively, early nutritional intervention (individualized nutritional counseling and oral supplements or enteral nutrition) or standard practice (general nutrition counseling). However, the percentage of patients who had radiotherapy breaks >5 days for toxicity was significantly lower in the early intervention group than in the standard practice group as well as the number of days of radiotherapy delayed for toxicity and the frequency of hospitalization. The study of Valentini *et al.* [29] has shown that, in patients with head and neck cancer receiving CRT, nutritional counseling combined with ONS was associated with relatively low CRT-related toxicity and with a percentage of patients interrupting anti-neoplastic treatment for 6 or more days lower than 30%.

Taken together, all these data support the concept, suggested by some authors [22,27], that head and neck cancer patients undergoing CRT need early and regular nutritional assessment and interventions during treatment and that dieticians need to adapt to the needs of each patient and provide individualized care. This is particularly true in patients with diabetes, which are not uncommon in such population.

## 4. Enteral Nutrition via Nasogastric Tube or Gastrostomy

International guidelines also suggest that if an obstructing head and neck cancer interferes with swallowing, enteral nutrition (EN) should be delivered by tube [20]. Tube feeding is also suggested if severe local mucositis is expected, which might interfere with swallowing, e.g., in radio-chemotherapy regimens, including radiation of throat [20].

Tube feeding can either be delivered via the nasogastric tube (NG) or percutaneous gastrostomy (PEG). Because of radiation induced oral and esophageal mucositis, PEG may be preferred and it has been demonstrated clearly that early and appropriate supplementary enteral nutrition via a PEG system is more effective than oral nutrition alone in those cases in which the patient undergoes several weeks of chemotherapy/radiotherapy [30]. However, PEG has a rate of complications that is estimated to be in the range 8%–30%, including local wound infection, occlusion of the tube, leakage from the tube, cellulitis, eczema or hypergranulation tissue [30].

Unfortunately, only three studies have compared NG and PEG in terms of nutritional outcomes, complications, and radiation treatment interruption (Table 2). Of these, two studies were retrospective and one prospective. In the study of Magnè *et al.* [31], 50 HNC patients were managed by PEG and 40 by NG. The feeding methods were found to be equally effective at maintaining body weight and body mass index at time 1 (three weeks) and at time 2 (six weeks). In the study of Mekhail *et al.* [32], NG tubes were placed in 29 patients and PEG in 62. PEG patients had more dysphagia at three months (59% *vs.* 30%, respectively; *p* = 0.015) and at six months (30% *vs.* 8%, respectively; *p* = 0.029) than NG patients. The median tube duration was 28 weeks for PEG patients compared with eight weeks for NG patients, (*p <* 0.001). Twenty-three percent of PEG patients needed pharyngo-esophageal dilatation compared with 4% of NG patients (*p* = 0.022). In the prospective study of Corry *et al.* [33], there were 32 PEG and 73 NGT patients. PEG patients sustained significantly less weight loss at six weeks post-treatment (median 0.8 kg gain *vs.* 3.7 kg loss, *p <* 0.001), but had a high insertion site infection rate (41%), longer median duration of use (146 *vs.* 57 days, *p <* 0.001), and more grade 3 dysphagia in disease-free survivors at six months (25% *vs.* 8%, *p* = 0.07). Patient self-assessed general physical condition and overall quality of life scores were similar in both groups. Overall costs were significantly higher for PEG patients. At six months post-treatment, there was no significant difference between the NGT and PEG groups in complete response at the primary site, weight, dysphagia grade 3 or performance status. Thirty-five percent of evaluable patients in the NGT group (18/52) had ≥10% loss of their body weight compared to 13% (3/23) in the PEG group (*p =* 0.09).

**Table 2:** Enteral feeding in head and neck cancer patients receiving chemoradiotherapy (CRT): comparison of nasogastric tube (NGT) and percutaneous gastrostomy (PEG). WL, weight loss; QOL, quality of life.

Author	Type of Study	Number of Patients	Cancer Therapy	Nutritional Outcome	Interruption of RT	Other Outcomes
Magnè *et al.*, 2001 [31]	Retrospective	PEG: 50 pts; NGT: 40 pts	CRT	Weight and BMI comparable at week 3 and 6	Not assessed	Better QOL with PEG
Mekhail *et al.*, 2001[32]	Retrospective	PEG: 62 pts; NGT: 29 pts	CRT	Not assessed	Not assessed	Dysphagia more persistent with PEG at 3 and 6 months; By 12 months, difference disappeared
Corry *et al.*, 2009 [33]	Prospective	PEG: 32 pts; NGT: 73 pts	CRT	WL (kg) at 6 weeks: PEG = +0.8 *vs.* NGT = −3.7; *p <* 0.001; WL (kg) at 6 months: PEG = +1 *vs.* NGT = −4.3; *p =* 0.04	Not assessed	PEG patients: high insertion site infection rate (41%), longer duration of use (146 *vs.* 57 days, *p* < 0.001), more grade 3 dysphagia at 6 months; higher costs

Interestingly, long-term swallow function after chemoradiotherapy for head and neck cancer seems to be similar in patients receiving prophylactic gastrostomy and nasogastric tube [34].

Little is known about the number of hospitalizations as well as the costs of the two different feeding approaches. In the study of Corry *et al.* [33], the number of days of hospitalization and costs in the PEG group were significantly higher than in the NGT group. However, if it is considered that PEG is now placed without hospitalization, it is possible that the cost consistently decrease significantly.

It seems that there is not sufficient evidence to determine the optimal method of enteral feeding for patients with head and neck cancer receiving chemoradiotherapy. Further trials comparing the two methods of enteral feeding and including an appropriate number of patients are required.

## 5. Prophylactic Nutritional Support

In the last decade, the prophylactic feeding (P-FT) through NGT or PEG, before beginning CRT, to prevent weight loss, reduce dehydration and hospitalizations, and avoid treatment breaks has become relatively common. Alternatively, patients are supported with oral nutritional supplements and, when it is impossible to maintain nutritional requirements, enteral feeding via a NGT or PEG is started (reactive feeding; R-FT).

Numerous studies have compared these two approaches as detailed in Table 3. Six studies were retrospective and two prospective, randomized [35,36,37,38,39,40,41,42]. In the majority of these studies, the nutritional outcome was similar in patients receiving prophylactic and reactive feeding. The number of interruptions of anti-cancer treatment was not assessed in two studies and did not differ significantly in five studies. In the study of Lewis *et al.*, patients with P-FT completed a higher proportion of chemotherapy cycles compared to no-FT (*p =* 0.002) and RFT (*p* < 0.001). When assessed, overall and disease-free survival were similar in the different groups of nutritional treatment. One study has shown that quality of life at six months was significantly higher in the group receiving systematic prophylactic gastrostomy [43].

It seems that prophylactic feeding, compared to reactive feeding (patients are supported with oral nutritional supplements and when it is impossible to maintain nutritional requirements enteral feeding via a NGT or PEG is started), does not offer significant advantages in terms of nutritional outcomes, interruptions of radiotherapy and survival. However, considering the limited number of prospective, randomized studies, definitive conclusions cannot be drawn and it is desirable that further investigations will be conducted on this issue in the next future.

Interestingly, Baschnagel *et al.* [44] have recently shown that there was no difference in the PEG tube dependence rates between PEG placed prophylactically *vs.* reactively. However, patients who received a PEG tube reactively had a significantly higher stricture rate and aspiration rate compared to the prophylactic group. In addition, there were significantly fewer hospitalizations in the prophylactic group compared to the reactive group. Overall, when accounting for both PEG placement and hospitalizations, the prophylactic approach was found to be more cost effective.

In 2013, Hughes *et al.* [45] retrospectively examined the data of HNC patients, who underwent CRT for the years before (2005) and after (2007) implementation of internal guidelines, in terms of number of hospitalization and costs. Only five patients (6.5% of all patients treated) in the 2005 cohort received prophylactic gastrostomy tubes compared with 39 patients (44.3%) in the 2007 cohort. Patients in 2007 had significantly fewer hospital admissions, unexpected admissions, and a shorter mean duration of hospital stay in comparison with those in 2005.

Noteworthy, a recent retrospective study has identified independent risk factors (BMI >25, a tumor classification ≥3, a cumulative cisplatin dose of 200 mg/m^2^) associated with symptomatic requirement for the reactive placement of a PEG tube [46].

**Table 3:** Prophylactic feeding in head and neck cancer patients receiving chemoradiotherapy (CRT). P-PEG, prophylactic percutaneous gastrostomy; R-PEG, reactive percutaneous gastrostomy. No-FT, no feeding tube; NGT, nasogastric tube; NC, nutritional counselling; ONS, oral nutritional supplements.

Author	Type of Study	Cancer Therapy	Number of Patients	Nutritional Treatment	Nutritional Outcome	Interruption of RT	Survival
Salas *et al.*, 2009 [43]	Randomized trial	CRT	39	P-PEG: 21 pts; R-PEG: 18 pts	Similar decrease of BMI at 6 months in the two groups	Not assessed	Survival not assessed. Better QOL at 6 months in the P-PEG group
Nugent *et al.*, 2010 [41]	Retrospective	CRT	76	ONS: 26 pts; NGT: 18 pts; P-PEG: 21 pts; R-PEG: 11 pts	WL% at end of treatment:ONS: 6.1NG-tube: 8.5 P-PEG: 4.6; T-PEG:8.7; (*p =* NS)	No differences between the groups	Not assessed
Chen *et al.*, 2010 [42]	Retrospective	CRT	120	Control: 20 pts; P-PEG: 70 pts	WL% at end of treatment: Control: 14; P-PEG: 8 (*p <* 0.001); WL% at 3 months: Control: 8P-PEG: 5; (*p =* 0.34)	No differences between the groups (*p =* 0.54)	No significant differences in the 3-year overall and disease-free survival
Silander *et al.*, 2012 [40]	Randomized trial	CRT	134	NC (+NGT): 70 pts; P-PEG: 64 pts	Same proportion of patients who had a 10 % weight loss at 3, 6 and 12 months	No differences between the groups (*p* = 0.08).	No differences in 2-year survival between the groups (*p =* 0.40)
Williams *et al.*, 2012 [39]	Retrospective	CRT	104	NGT: 21 pts; P-PEG: 71 pts; R-PEG: 12 pts	No differences in weight loss at the end of treatment and at 6 months post-radiotherapy (*p =* 0.23).	No differences between the groups (*p* = 0.47).	No significant differences in disease free and overall survival between the groups (*p* = 0.90 and *p* = 0.13, respectively)
Olson *et al.*, 2013 [38]	Retrospective	CRT	445	Center A, prefers R-PEG; Center B, prefers P-PEG:	Same % of patients with 10% weight loss at 1 year in the two centers	Not assessed	No significant differences in the overall survival
Lewis *et al.*, 2013 [37]	Retrospective	CRT	109	Control: 50 pts; P-PEG:25 pts; R-PEG: 34 pts	Weight loss (%): Control: 15.2; P-PEG: 2.4; R-PEG: 10.4	Patients with P- PEG completed a higher proportion of chemotherapy cycles compared to control (*p =* 0.002) and R- PEG (*p* <0.001).	Not assessed
Kramer *et al.*, 2014 [36]	Retrospective	CRT	74	P-PEG: 56 pts; R-PEG: 300 pts	No difference in weight loss (%) at 2, 6, 12 months.	Not assessed.	No difference in survival or disease control

## 6. Conclusions

Head and neck cancer patients undergoing chemoradiotherapy are at risk of malnutrition before and during treatment. Nutritional counseling and oral nutritional supplements should be used to increase dietary intake and to prevent therapy-associated weight loss and interruption of radiation therapy. If obstructing cancer and/or mucositis interfere with swallowing, enteral nutrition should be delivered by tube. However, it seems that there is not sufficient evidence to determine the optimal method of enteral feeding. Prophylactic feeding through nasogastric tube or percutaneous gastrostomy to prevent weight loss, reduce dehydration and hospitalizations, and avoid treatment breaks has become relatively common. However, compared to reactive feeding (patients are supported with oral nutritional supplements and when it is impossible to maintain nutritional requirements enteral feeding via a NGT or PEG is started), prophylactic feeding does not offer advantages in terms of nutritional outcomes, interruptions of radiotherapy and survival.

Overall, it seems that further adequate prospective, randomized studies are needed to define the better nutritional intervention in head and neck cancer patients undergoing chemoradiotherapy and to eventually change the current practice, having in mind that the nutritional treatment of these patients is complex and requires a multidisciplinary approach.
